# Gain and loss of elongation factor genes in green algae

**DOI:** 10.1186/1471-2148-9-39

**Published:** 2009-02-12

**Authors:** Ellen Cocquyt, Heroen Verbruggen, Frederik Leliaert, Frederick W Zechman, Koen Sabbe, Olivier De Clerck

**Affiliations:** 1 Phycology Research Group and Center for Molecular Phylogenetics and Evolution, Ghent University, Krijgslaan 281 S8, 9000 Ghent, Belgium; 2 Department of Biology, California State University, Fresno, 2555 East San Ramon Avenue, Fresno, California 93740, USA; 3 Protistology and Aquatic Ecology Research Group, Ghent University, Krijgslaan 281 S8, 9000 Ghent, Belgium

## Abstract

**Background:**

Two key genes of the translational apparatus, elongation factor-1 alpha (EF-1α) and elongation factor-like (EFL) have an almost mutually exclusive distribution in eukaryotes. In the green plant lineage, the Chlorophyta encode EFL except *Acetabularia *where EF-1α is found, and the Streptophyta possess EF-1α except *Mesostigma*, which has EFL. These results raise questions about evolutionary patterns of gain and loss of EF-1α and EFL. A previous study launched the hypothesis that EF-1α was the primitive state and that EFL was gained once in the ancestor of the green plants, followed by differential loss of EF-1α or EFL in the principal clades of the Viridiplantae. In order to gain more insight in the distribution of EF-1α and EFL in green plants and test this hypothesis we screened the presence of the genes in a large sample of green algae and analyzed their gain-loss dynamics in a maximum likelihood framework using continuous-time Markov models.

**Results:**

Within the Chlorophyta, EF-1α is shown to be present in three ulvophycean orders (i.e., Dasycladales, Bryopsidales, Siphonocladales) and the genus *Ignatius*. Models describing gene gain-loss dynamics revealed that the presence of EF-1α, EFL or both genes along the backbone of the green plant phylogeny is highly uncertain due to sensitivity to branch lengths and lack of prior knowledge about ancestral states or rates of gene gain and loss. Model refinements based on insights gained from the EF-1α phylogeny reduce uncertainty but still imply several equally likely possibilities: a primitive EF-1α state with multiple independent EFL gains or coexistence of both genes in the ancestor of the Viridiplantae or Chlorophyta followed by differential loss of one or the other gene in the various lineages.

**Conclusion:**

EF-1α is much more common among green algae than previously thought. The mutually exclusive distribution of EF-1α and EFL is confirmed in a large sample of green plants. Hypotheses about the gain-loss dynamics of elongation factor genes are hard to test analytically due to a relatively flat likelihood surface, even if prior knowledge is incorporated. Phylogenetic analysis of EFL genes indicates misinterpretations in the recent literature due to uncertainty regarding the root position.

## Background

Elongation factor-1 alpha (EF-1α) is a core element of the translation apparatus and member of the GTPase protein family. The gene has been widely used as a phylogenetic marker in eukaryotes; either to resolve their early evolution [e.g., [1,2]] or more recent phylogenetic patterns [e.g., [3-7]]. The evolutionary history of genes used for such inferences should closely match that of the organisms and not be affected by ancient paralogy or lateral gene transfer [8]. A gene related to but clearly distinguishable from EF-1α, called elongation factor-like (EFL), appears to substitute EF-1α in a scattered pattern: several unrelated eukaryote lineages have representatives that encode EFL and others that possess EF-1α. The EFL and EF-1α genes are mutually exclusive in all but two organisms: the zygomycete fungus *Basidiobolus *and the diatom *Thalassiosira *[9,10]. Although EFL is found in several eukaryotic lineages, EF-1α is thought to be the most abundant of both [11]. So far, EFL has been reported in chromalveolates (*Perkinsus*, dinoflagellates, diatoms, haptophytes, cryptophytes), the plant lineage (green and red algae), rhizarians (cercozoans, foraminifera), unikonts (some Fungi and choanozoans) and centrohelids [8,10,12-14]].

The mutually exclusive distribution of EF-1α and EFL suggests similar functionality. The main function of EF-1α is translation initiation and termination, by delivering aminoacyl tRNAs to the ribosomes [15]. Other functions include interactions with cytoskeletal proteins: transfer, immobilization and translation of mRNA and involvement in the ubiquitine-dependent proteolytic system, as such forming an intriguing link between protein synthesis and degradation [15]. In contrast, the function of EFL is barely known. It is assumed to have a translational function because the putative EF-1β, aa-tRNA, and GTP/GDP binding sites do not differ between EF-1α and EFL [8]. Based on a reverse transcriptase quantitative PCR assay in the diatom *Thalassiosira*, which possesses both genes, it was proposed that EFL had a translation function while EF-1α performed the auxiliary functions [10].

The apparently scattered distribution of EFL across eukaryotes raises questions about the gain-loss patterns of genes with an important role in the cell. This mutually exclusive and seemingly scattered distribution can be explained by two different mechanisms: ancient paralogy and lateral gene transfer. Ancient paralogy was considered unlikely because this would imply that both genes were present in ancestral eukaryotic genomes during extended periods of evolutionary history while the genes rarely coexist in extant species [8]. Furthermore, a prolonged coexistence of both genes in early eukaryotes would have likely resulted in either functional divergence or pseudogene formation of one or the other copy [16], as is suggested for EFL and EF-1α coexisting in the diatom *Thalassiosira *[10]. Keeling and Inagaki [8] proposed lateral gene transfer of the EFL gene between eukaryotic lineages as the most likely explanation for the scattered distribution of both genes.

In the green plants (Viridiplantae), EF-1α and EFL seem to show a mutually exclusive distribution. Of the two major green plant lineages, the Chlorophyta were shown to have EFL with the exception of *Acetabularia *where EF-1α is found, and the Streptophyta were shown to possess EF-1α with the exception of *Mesostigma*, which has EFL [13]. Noble et al. [13] proposed the hypothesis that EFL was introduced once in the ancestor of the green lineage, followed by differential loss of EF-1α or EFL in the principal clades of the Viridiplantae (i.e., Streptophyta and Chlorophyta).

The goals of the present study are to extend our knowledge of the distribution pattern of EF-1α and EFL in the green algae and investigate patterns of gain and loss of these key genes of the translational apparatus. We applied a RT-PCR and sequencing-based screening approach across a broad spectrum of green algae, with emphasis on the ulvophycean relatives of *Acetabularia*. To test the hypothesis of Noble et al. [13], we modeled patterns of gene gain and loss. To this goal, a reference phylogeny based on three commonly used loci was inferred, and gain-loss dynamics of EFL and EF-1α were optimized along this phylogeny using continuous-time Markov models.

## Results and discussion

### Distribution of elongation factors in the green algae

**Figure 1:**
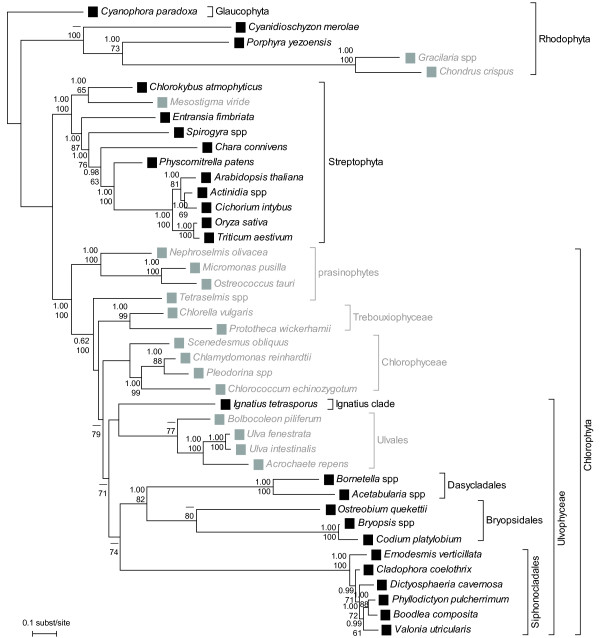
**Distribution of EF-1α and EFL in the green plants**. The type of elongation factor is indicated with black (EF-1α) or gray (EFL) squares. The reference phylogeny was obtained by Bayesian phylogenetic inference of nuclear SSU rDNA and the plastid genes *rbc*L and *atp*B. Numbers at nodes indicate posterior probabilities (top) and ML bootstrap values (bottom); values below respectively 0.9 and 50 are not shown.

**Figure 2:**
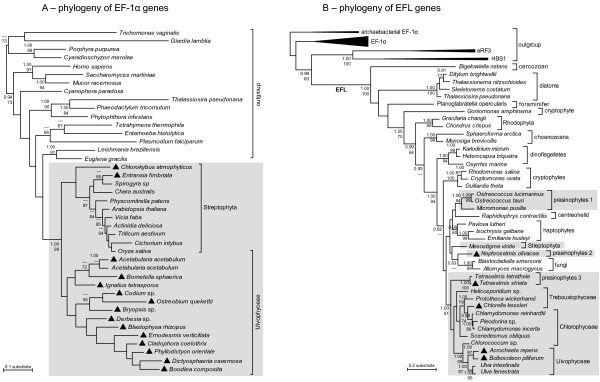
**Phylogenies inferred from EF-1α and EFL amino-acid sequences with Bayesian techniques**. Sequences belonging to the green plant lineage are in gray boxes. Whereas all green plant EF-1α sequences group in a single clade, the green plant EFL sequences seem to form separate lineages. Sequences generated for this study are indicated with triangles. Numbers at nodes indicate posterior probabilities (top) and ML bootstrap values (bottom); values below respectively 0.9 and 50 are not shown.

EF-1α sequences were retrieved from streptophytes *Entransia *(Klebsormidiophyceae) and *Chlorokybus *(Chlorokybophyceae), confirming previous observations that all Streptophyta except *Mesostigma *have EF-1α. We found EFL sequences in *Chlorella *(Trebouxiophyceae), *Acrochaete *and *Bolbocoleon *(Ulvophyceae), *Nephroselmis *and *Tetraselmis striata *(prasinophytes), further confirming the formerly established distribution pattern within the Chlorophyta. We reaffirmed the presence of EFL in *Chlamydomonas *and *Scenedesmus *(Chlorophyceae), *Ulva intestinalis *and *U. fenestra *(Ulvophyceae) and *Ostreococcus *(prasinophytes), previously shown by Noble et al. [13]. In addition to *Acetabularia*, EF-1α was discovered in representatives of the ulvophycean orders Dasycladales (*Bornetella*), Bryopsidales (*Blastophysa*, *Bryopsis*, *Codium*, *Derbesia*, *Ostreobium*), Siphonocladales (*Boodlea*, *Cladophora*, *Dictyosphaeria*, *Ernodesmis*, *Phyllodictyon*) and in *Ignatius *(see Figures 1 and 2). The RT-PCR approach did not reveal the presence of both genes in any of the screened species despite the fact that our primers could amplify the target genes across the Viridiplantae. Our RT-PCR experiments on two species whose genomes have been sequenced (*Chlamydomonas *and *Ostreococcus*) yielded a single gene for each species, a result in compliance with the knowledge derived from their genome sequences [17].

The reference phylogeny, inferred from a DNA matrix consisting of 72 taxa representing all major plant lineages and three loci (SSU rDNA, *rbc*L and *atp*B), is in accordance with recent phylogenetic studies, including the position of *Mesostigma *within the Streptophyta [18,19]. Figure 1 shows the phylogenetic relationships among the taxa for which we have information on elongation factors; the full 72-taxon phylogeny can be found as an online supplement [see Additional file 1]. Even though the tree shows improved resolution from previous studies, large parts of the backbone remained poorly resolved. In order to obtain a solid hypothesis of green algal evolution, much additional sequence data may have to be gathered. The occurrence of EF-1α and EFL in terminal taxa was plotted on the reference phylogeny in Figure 1. *Mesostigma *is the only streptophyte which encodes EFL. Within the chlorophytan class Ulvophyceae, the order Ulvales possesses EFL whereas the other orders encode EF-1α (Dasycladales, Siphonocladales, Bryopsidales and *Ignatius*).

### Phylogenies of EF-1α and EFL

All green plant EF-1α sequences form a monophyletic group clearly differentiated from EF-1α sequences of a variety of other eukaryotes (Figure 2A). Even though the Viridiplantae form a strongly supported group, resolution among and within Streptophyta and Chlorophyta is generally low, which could in part be due to some short EF-1α sequences included in the analysis.

In contrast, green plant EFL genes do not form a monophyletic lineage (Figure 2B). Although the backbone of the phylogeny is moderately resolved, monophyly of green plant EFL genes is unlikely because it is not observed in the MCMC output (zero posterior probability). EFL sequences of the Viridiplantae can be found in several clades. The chlorophytes, trebouxiophytes, ulvophytes and prasinophyte *Tetraselmis *form a single monophyletic group. The other prasinophyte EFL sequences form two separate groups. The last clade consists of the streptophyte *Mesostigma*.

To obtain an accurate root position for our EFL tree, we included related subfamilies of the GTPase translation factor superfamily: EF-1α, eukaryotic release factor 3 (eRF3), heat shock protein 70 subfamily B suppressor (HBS1) and archaebacterial EF-1α sequences in our analyses. In accordance with Keeling and Inagaki [8], the tree is rooted with archaebacterial EF-1α sequences. All analyses (Bayesian and ML) resulted in a phylogeny very similar to the one shown in Figure 2B, the complete phylogeny with all related subfamilies can be found as an online supplement [see Additional file 2]. This phylogeny shows seven EFL clades, with the following branching order: *Bigelowiella*, the diatoms, *Planoglabratella*, the cryptophyte *Goniomonas*, red algae, choanozoans, and a large clade containing the green plant lineage, chromalveolates (dinoflagellates, haptophytes, cryptophytes), fungi and *Rhaphidiophrys *(Figures 2B). Deep branches generally received low statistical support, preventing strong conclusions about the relationship between the seven clades.

### Gain-loss dynamics

The scattered distribution of EF-1α and EFL in the green plant lineage is a remarkable phenomenon that raises questions about evolutionary patterns of gain and loss of both genes.

Noble et al. [13] proposed the hypothesis that EF-1α was present in the common ancestor of the plant lineage, followed by a single gain of EFL early in evolution of the green lineage and subsequent differential loss of one or the other gene in the various lineages. Our aim was to test this hypothesis by modeling gain-loss dynamics and inferring ancestral presence-absence patterns of both genes in a maximum likelihood framework. Gene gain and loss rates were estimated by maximum likelihood (ML) optimization, using a dataset of presence-absence patterns of EF-1α and EFL and a reference phylogeny derived from the Bayesian analysis of three commonly used loci (SSU nrDNA, *rbc*L and *atp*B).

**Figure 3:**
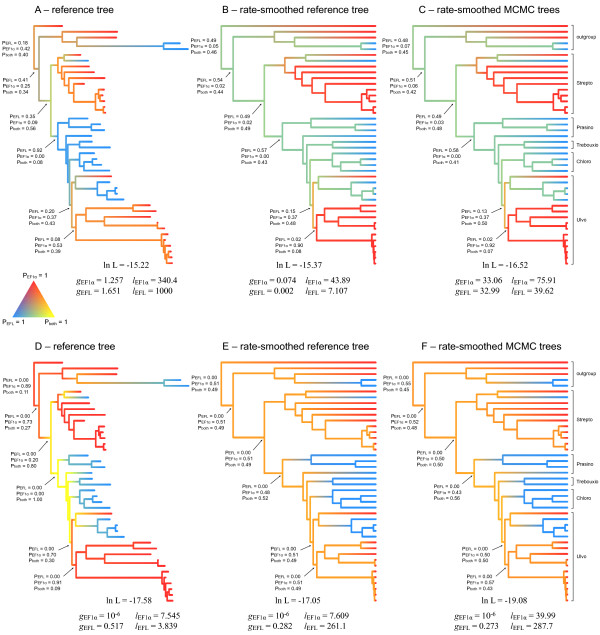
**Gain-loss dynamics of green algal elongation factor genes and their inferred presence in ancestral genomes**. Gain and loss rates, as well as the estimated probabilities for presence of the genes in ancestral genomes are given for a variety of analysis conditions. Panels A-C show the outcome of models in which EF-1α and EFL gain and loss rates were not constrained. In panels D-F, the gain rate of EF-1α was constrained to be 10^-6^. Colors were used to visualize estimated probabilities for presence of genes along the tree. Red indicates a high probability for EF-1α, blue marks a high probability of EFL and yellow stands for a high probability of the presence of both genes. Intermediate colors indicate uncertainty.

A first analysis, based on the reference tree, shows uncertain character state probabilities along the backbone of the Viridiplantae and suggests a loss of EF-1α in early Chlorophyta evolution and regain in some Ulvophyceae (Figure 3A). Because branch lengths play a crucial role in model optimization, the analysis was repeated on an alternative version of the reference tree in which branch lengths were transformed using a rate smoothing approach. Since our tree deviates from the molecular clock, we performed rate smoothing to obtain branch lengths roughly proportional to time. Rate smoothing techniques relax the assumption of constant rates of evolution throughout the tree: differences in rates of molecular evolution are smoothed out by assuming that evolutionary rates change gradually throughout the phylogeny. The result is an ultrametric tree in which branch lengths are roughly proportional to evolutionary time instead of amounts of molecular evolution. Modeling gain-loss dynamics of elongation factor genes along the rate-smoothed tree yields results that strongly deviate from those obtained with the original reference tree: probabilities of the character states along the major part of backbone are now around 50% for EFL and around 50% for the presence of both genes (Figure 3B). Subsequently, an additional level of realism was introduced by taking phylogenetic uncertainty into account because several nodes in the reference tree are poorly supported. To this goal, all post-burnin MCMC trees were rate-smoothed and analyzed individually. The results were summarized on the rate-smoothed reference tree. Taking phylogenetic uncertainty into consideration had a minor influence on the probabilities of the characters states (Figure 3C).

Although the exact numbers differ between analyses, gene gain rates were always lower than gene loss rates, reinforcing the notion that gene transfers are rare events in comparison to losses of redundant genes [20]. Whereas the analysis based on the original reference tree returned faster gain and loss rates for EFL than for EF-1α, analyses based on rate-smoothed trees (including MCMC trees) suggested the inverse, marking the sensitivity of Markov models to the unit of operational time.

From these results, it seems fair to conclude that there is major uncertainty about the ancestral states for a variety of reasons, including sensitivity to branch lengths and lack of prior knowledge about ancestral states or rates of gene gain and loss. Considering that the ancestors must have had either EF-1α, EFL or both genes opens perspectives for hypothesis comparison in a likelihood framework. Additionally, information about rates of gene gain and loss could be gleaned from the EF-1α and EFL phylogenies.

Analyses constrained with various hypotheses about ancestral gene content resulted in a confidence set of 8 trees that differ extensively [see Additional file 3]. The fact that strongly different hypotheses are also present in the confidence set denotes that the likelihood surface is too flat to draw firm conclusions in favor of one or another hypothesis.

The last option to reduce uncertainty is to inform the Markov models with information on gains and losses gleaned from the EF-1α and EFL trees [cf. [20]]. Because green plant EF-1α sequences form a monophyletic and strongly supported lineage, it seems fair to assume vertical descent of EF-1α throughout the Viridiplantae. This knowledge can be introduced in our Markov model by setting a very low gain rate of EF-1α. If the analysis is constrained in this way, both EFL and EF-1α were inferred to be present along the backbone of the Viridiplantae in the original reference tree (Figure 3D) and a 50/50 probability for the presence of EF-1α or both genes was obtained in the rate-smoothed trees (Figures 3E–F). Comparison of hypotheses about ancestral gene content constrained with a very low EF-1α gain rate reduced the confidence set to 3 trees in which either EF-1α or both genes are present along the backbone [see Additional file 4]. The ML solution (hypothesis 122) assumes that only EF-1α was present along the backbone of the tree and consequently shows independent gains of EFL in *Mesostigma*, prasinophytes, Chlorophyceae, Trebouxiophyceae and Ulvales. An alternative scenario (hypothesis 123) in the confidence set has EF-1α at the base of the Viridiplantae, a gain of EFL in the ancestor of the Chlorophyta, and subsequent differential loss of one or the other gene in the various lineages. Information from the EFL phylogeny may provide clues for further distinction between either multiple transfers or ancient paralogy with subsequent losses.

The green EFL sequences form a highly supported clade together with dinoflagellates, cryptophytes, haptophytes, fungi and *Rhaphidiophrys*, suggesting lateral gene transfer of the EFL gene between these distant eukaryotic lineages [21,22]. Considering the ability of chromalveolates (i.e., dinoflagellates, cryptophytes and haptophytes) and *Raphidiophrys *to feed through phagocytosis [23] and the absence of this behavior in green algae, it would be tempting to assume that lateral gene transfer occurred from green algae to the dinoflagellates, cryptophytes, haptophytes and *Raphidiophrys *instead of the other way around. Phagotrophic eukaryotes have been shown to have elevated rates of lateral gene transfer [21,24] because this feeding mechanism enables them to continually recruit genes from engulfed prey [25]. Lateral gene transfers to fungi, although known to exist [26], would require a different explanation because neither phagotrophy nor endosymbiosis occur in fungi. However, in the light of this peripheral information, it would be tempting to conclude that both EF-1α and EFL essentially show vertical descent in green plants and that the observed mutually exclusive pattern of EFL and EF-1α sequences results from differential loss. In this scenario, lateral gene transfer must have occurred from green algal cells to other eukaryotic lineages.

In previous studies of functionally similar eukaryotic genes with mutually exclusive distributions, distinction between ancient paralogy with subsequent losses and multiple transfers was made based on two main criteria [11]. The first criterion states that if one gene dominates the tree and the other occurs in only a few lineages, multiple independent transfers should be regarded as the most probable explanation whereas equal representation would suggest common ancestry with subsequent differential loss. The second criterion is about the age of the taxa involved. If the mutually exclusive pattern occurs between closely related species, one can conclude common ancestry with subsequent losses. If the pattern is more ancient, multiple lateral transfers are a more probable explanation. It is obvious that such criteria are very difficult to apply in real situations. These difficulties can be overcome by taking a probabilistic angle on the problem and modeling gain-loss dynamics with continuous-time Markov models. This approach brings statistical rigor to the analysis of gene presence-absence patterns and has the potential to discriminate between the alternative scenarios of ancient paralogy with differential losses and multiple independent lateral transfers. Application of this technique to our dataset of green algal elongation factors revealed the difficulty of arriving at firm conclusions about ancient gene transfer events because of a relatively flat likelihood surface and, consequently, ambiguous probabilities for gene content at ancestral nodes. When informed with external information, the analyses allow somewhat more definitive conclusions.

### The broader eukaryotic picture

**Figure 4:**
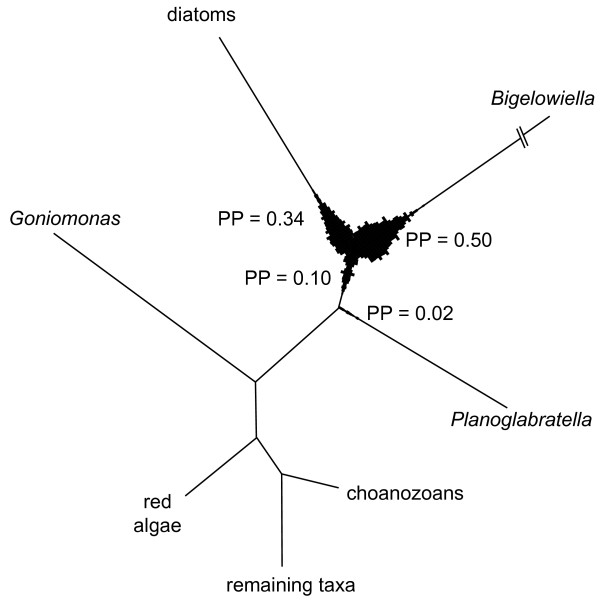
**Visualization of the posterior probability of rooting of the EFL tree**. The topology represents the unrooted topology of EFL genes. Branch width is proportional to the posterior probability that the outgroup, consisting of archaebacterial EF-1α, EF-1α, eRF3 and HBS1 sequences, attaches to the ingroup tree at that point. Numbers at branches represent the total posterior probability that the root is situated along the branch in question.

In addition to the information gained about elongation factor evolution in green algae, our results also highlight misinterpretations in recent literature on EFL evolution across the eukaryotes. Previous studies have not been explicit about whether or how their phylogenetic trees were rooted, but have drawn conclusions that require directionality in the tree. Kamikawa et al. [10] concluded that lateral gene transfer from a foraminifer (*Planoglabratella*) to the ancestor of the diatoms must have occurred because the diatom sequences were nested within the Rhizaria (foraminifera and cercozoans). In case their tree was unrooted, this conclusion is flawed due to a lack of directionality in the tree. In their presentation of the tree, choanozoans are used as one of the basal clades, probably because they were the earliest-branching lineage in the tree presented by Keeling and Inagaki [8]. Our EFL tree, which includes EF-1α, eRF3 and HBS1 sequences and is rooted with archaebacterial EF-1α sequences, indicates that the directionality inferred by Kamikawa et al. [10] is likely to be wrong. Our phylogram (Figures 2B) suggest that the root position of EFL lies on the branch leading towards the cercozoan *Bigelowiella*, but support is lacking for the basal relationships. A plot of the posterior distribution of root placements (Figure 4) illustrates the uncertainty about the root placement more clearly. It is evident from this plot that the choanozoans are not the oldest diverging lineage. This finding overturns the conclusion from Kamikawa et al. [10] because the nested position of the diatom EFL genes within the Rhizaria sequences can no longer be maintained. Our EFL phylogeny supports the presence of lateral gene transfer between eukaryotic lineages, however, the direction of lateral gene transfer is difficult to evaluate.

## Conclusion

The mutually exclusive nature of EF-1α and EFL is confirmed in a large sample of green algae. The Streptophyta possess EF-1α with the exception of *Mesostigma*, which has EFL. The Chlorophyta encode EFL with the exception of Dasycladales, Bryopsidales, Siphonocladales and *Ignatius*, where EF-1α is found. This result establishes EF-1α as a widespread gene among green algae.

Gain-loss models revealed that the probabilities of the presence of EF-1α, EFL or both genes along the backbone of the plant phylogeny are highly uncertain, and that a previously published hypothesis [13] is as likely as several other hypotheses. Model refinements based on insights gained from the EF-1α phylogeny were unable to distinguish between three possibilities: (1) multiple, independent gains of EFL throughout the plant lineage, (2) a single gain of EFL early in evolution of the plant lineage followed by differential loss, or (3) independent gains of EFL in *Mesostigma *and the ancestor of the Chlorophyta followed by differential loss of one or the other gene in the various lineages (Figure 3D–F and Additional file 4).

Further research into the gain-loss dynamics of elongation factors of green plants and eukaryotes in general is needed to come to more definitive conclusions about their evolution. First, the EFL phylogeny should be refined by obtaining full-length sequences for a set of relevant taxa to confirm or reject the presence of multiple independent green lineages in this tree. The use of codon models may help to achieve this [27]. An alternative approach would be to learn about the processes responsible for lateral transfer of elongation factors by studying their flanking regions for signature sequences of mobile elements [28,29]. Finally, studying gain-loss dynamics across a wider spectrum of eukaryotic supergroups should lead to more stable conclusions. In addition to yielding more precise parameter estimates for gene gain and loss rates, a eukaryote-wide study would allow the use of more specific models for lateral gene transfer because both donor and recipient lineages would be present in the analysis [30-32]. It remains an enigma that the evolution of elongation factors, genes crucial for cell functioning, is marked by such complex gain-loss patterns.

## Methods

### Algal strains

Algal strain information is provided as additional material online [see Additional file 5]. All cultures were grown at 18°C, except Dasycladales, Siphonocladales and *Derbesia *(23°C). Cool white fluorescent lamps were used for a 12/12 h light/dark cycle. Marine cultures were maintained in f/2 medium and freshwater cultures in Bold's Basal Medium [33].

### RNA isolation and cDNA library construction of *Cladophora coelothrix*

Total RNA was extracted with a RNeasy Plant Mini Kit (Qiagen Benelux b.v., Venlo, the Netherlands) or a NucleoSpin^® ^RNA Plant kit (Macherey-Nagel GmbH & Co. KG, Düren, Germany) according to the manufacturer's instructions, including a DNase step to eliminate genomic DNA contamination. RNA quality was checked on a 1% agarose gel (made with 1× TAE diluted in 0,1% DEPC water). RNA concentration and purity were measured in a spectrophotometer at 260 and 280 nm according to standard methods [34].

Approximately 30 μg of total RNA of *Cladophora coelothrix *was extracted as described above. A standard cDNA library was constructed by VERTIS Biotechnologie AG (Freising, Germany). An EF-1α sequence of 624 bp was obtained by sequencing randomly picked clones.

### Reverse Transcriptase and Polymerase Chain Reaction

cDNA construction was performed with the Omniscript RT kit (Qiagen) and oligodT primers according to the manufacturer's instructions; the reaction was incubated for several hours at 37°C.

Primers were designed to fit the most conserved regions of EF-1α and EFL sequences across Viridiplantae. Primers for EF-1α were based upon aligned GenBank sequences from green algae (*Acetabularia *and *Chara*) and land plants, completed with our *Cladophora coelothrix *cDNA sequence (EF-1α-F: 5'-GGC CAT CTT ATC TAC AAG CTT GGC GG-3' and EF-1α-R: 5'-CCA GGA GCA TCA ATC ACG GTG CAG-3'). EFL primers were adapted from Noble et al. [13] (EFL-F: 5'-TCC ATY GTS ATY TGC GGN CAY GTC GA-3' and EFL-R: 5'-CTT GAT GTT CAT RCC RAC RTT GTC RCC-3'). PCR amplification was performed with the following reaction mixture: 1 μl of cDNA, 2.5 μl of 10× Buffer (Qiagen), 0.5 μl dNTP's (10 mM), 0.5 μl MgCl (25 mM, Qiagen), 0.5 μl of each primer (10 μm), 0.25 μl BSA (10 μg/μl), 18.125 μl sterilized MilliQ water and 0.125 μl Taq polymerase (5 U/μl, Qiagen). The amplification profile consisted of an initial denaturation of 2 min at 94°C, followed by 35 cycles of 30 s at 94°C, 30 s at 55°C and 45 s at 72°C and a final extension of 10 min at 72°C. Products of expected size (300 bp for EF-1α and 900 bp for EFL) were either sequenced directly or cloned and sequenced.

### Cloning and sequencing

PCR products were first sequenced with the forward primer with an Applied Biosystems 3130xl. Sequences were blasted against the GenBank protein database (blastx), to check for potential bacterial contaminants. Sequences without ambiguous base calls yielding a significant hit for Viridiplantae were further sequenced with the reverse primer. When ambiguous base calls were present in sequences, samples were cloned if the rough sequence gave a significant blastx hit for Viridiplantae. Cloning was performed with the pGEM^®^-T Vector System (Promega Benelux b.v., Leiden, the Netherlands) according to the manufacturer's instructions. After ligation, transformation and incubation, the white colonies were transferred to 15 μl double distilled water, boiled for 10 minutes to lyse cells and subsequently centrifuged to pellet the cells walls and allow harvest of the DNA in the liquid phase. Between three and five clones were PCR amplified and sequenced with the vector specific primers T7 and SP6 following the protocol described above. Cloning showed minor polymorphisms that most likely represent different alleles.

### Alignments and phylogenetic analysis of EF-1α and EFL

Sequences [see Additional file 6] were assembled with AutoAssembler 1.4.0 (ABI Prism, Perkin Elmer, Foster City, CA, USA) and aligned manually for both genes separately, resulting in EF-1α and EFL alignments of 1374 and 1653 bp, respectively [see Additional file 7]. Sequences generated with our primers begin in the N-terminal part the of the gene and are 900 bp for EFL and 150–300 bp for EF-1α. We included eukaryotic EF-1α, eRF3 and HBS1 sequences as well as archeabacterial EF-1α sequences to serve as outgroups for the EFL phylogeny [8]. Due to the large divergences between EFL and the other genes, Gblocks was run to remove ambiguously aligned regions [35]. We ran Gblocks v.0.91b, allowing smaller final blocks, gap positions within the final blocks and less strict flanking positions, resulting in an alignment of 358 amino acids [see Additional file 7]. The resulting EFL and EF-1α alignments were subjected to Bayesian phylogenetic inference with MrBayes 3.1.2 [36] using the model suggested by ProtTest 1.4 [37] (WAG with among site rate heterogeneity: gamma distribution with 8 categories). Two parallel runs, each consisting of four incrementally heated chains were run for 1,000,000 generations, sampling every 1,000 generations. Convergence of log-likelihoods was assessed in Tracer v1.4 [38]. A burnin sample of 100 trees was removed before constructing the majority rule consensus tree for each of the genes. Maximum likelihood phylogenies were inferred for EF-1α and EFL with Treefinder [39]. The analyses were based on amino acid sequences and used a WAG model with among site rate heterogeneity (gamma distribution with 8 categories). One thousand non-parametric bootstrap trees were inferred. Bootstrap values were summarized with consense from the Phylip package [40] and plotted onto the Bayesian consensus tree.

### Phylogeny of the green plants: SSU rDNA, *rbc*L and *atp*B

A reference phylogeny of green plants for which the presence of EF-1α or EFL is known was constructed using three commonly used phylogenetic markers: nuclear SSU rDNA and plastid *atp*B and *rbc*L genes and rooted with red algae and a glaucophyte. To obtain an even species distribution and consequently a better phylogenetic tree [41], many additional species were included in the phylogenetic analysis [see Additional file 7]. Sequences were retrieved from GenBank and aligned with our own sequences [see Additional file 6]. DNA was extracted using a standard CTAB method. PCR conditions followed standard protocol. Primers were based on other publications: SSU rDNA [42,43], *rbc*L [44] and *atp*B [45,46]. The *rbc*L and *atp*B sequences were aligned by eye. The SSU rDNA sequences were aligned based on their RNA secondary structure with DCSE [see Additional file 8].

The model selection procedure [see Additional file 8] proposed eight partitions: *atp*B and *rbc*L genes were partitioned into codon positions (6 partitions) and the SSU rDNA was partitioned into RNA loops and stems (2 partitions). Bayesian phylogenetic inference was carried out using a GTR model with gamma distribution and 8 rate categories per partition (all parameters unlinked) and rate multipliers to accommodate rate differences among partitions. Two parallel runs, each consisting of four incrementally heated chains were run for 5,000,000 generations, sampling every 1,000 generations. Convergence of log-likelihoods was assessed in Tracer v1.4 [38]. A burnin sample of 3,000 trees was removed before constructing the majority rule consensus tree. A maximum likelihood phylogeny was inferred with Treefinder [39]. The analysis used a GTR model with among site rate heterogeneity (gamma distribution with 8 categories). One thousand non-parametric bootstrap trees were inferred. Bootstrap values were summarized with consense from the Phylip package [40] and plotted onto the Bayesian consensus tree.

To obtain trees suitable for modeling gene gain and loss, the Bayesian consensus tree and the complete post-burnin set of trees were pruned to the set of species for which the type of elongation factor is known using the APE package [47]. Because our data deviate from the molecular clock, we performed rate smoothing to obtain branch lengths that are roughly proportional to time. We used the penalized likelihood method [48] implemented in the r8s program [49], with a log-additive penalty and a smoothing value of 2, which was the optimal value in cross-validation [48]. PL rate smoothing was applied to the Bayesian consensus tree as well as the post-burnin set of MCMC trees.

### Modeling gene gain and loss

If the presence of EF-1α and EFL are coded as two binary characters, their gain-loss dynamics can be modeled along a reference phylogeny using a continuous-time Markov model. Given the likely dependency of gain and loss between EF-1α and EFL, a model designed to study interdependent trait evolution was used [50]. The rate matrix of this model is given by:

(1) $$Q_{\text{D}}=\begin{array}{c} \begin{array}{cc} & \begin{array}{cccc} 0,0 & 0,1 & 1,0 & 1,1 \end{array} \end{array}\\ \begin{array}{cc} \begin{array}{c} 0,0\\ 0,1\\ 1,0\\ 1,1 \end{array} & \left(\begin{array}{cccc} \cdot & q_{12} & q_{13} & 0\\ q_{21} & \cdot & 0 & q_{24}\\ q_{31} & 0 & \cdot & q_{34}\\ 0 & q_{42} & q_{43} & \cdot \end{array}\right) \end{array} \end{array}$$

where (0,0) indicates the absence of both genes from the genome, (0,1) and (1,0) denote the presence of EFL and EF-1α, respectively, and (1,1) is the state where both genes are present in the genome. Different q's indicate relative rates of the respective changes in gene content. Transitions that require more than one event (e.g. 1,0 → 0,1) are not allowed to occur as a single step in this model, the logic being that the probability of two traits changing at exactly the same time is negligible. This is consistent with the fact that transitions from EF-1α to EFL and vice versa should pass through a stage where both genes are present in the genome. The elements of the diagonal are determined by the requirement that each row sums to zero. Because the absence of both genes is likely to be lethal, the matrix was constrained by introducing a series of very low rates as follows:

(2) $$Q_{\text{D}}=\left(\begin{array}{cccc} \cdot & 10^{-6} & 10^{-6} & 0\\ 10^{-6} & \cdot & 0 & g_{\text{EF}1\alpha }\\ 10^{-6} & 0 & \cdot & g_{\text{EFL}}\\ 0 & l_{\text{EF}1\alpha } & l_{\text{EFL}} & \cdot \end{array}\right)$$

In this matrix, *g*_EF1α _and *g*_EFL _denote gain rates and *l*_EF1α _and *l*_EFL _loss rates. It must be noted that the model does not take gene duplications into account because our data provided no indications for the presence of such events.

The rate matrix (2) was specified as a special case of the "discrete dependent" model in BayesTraits [51]. The model parameters were estimated by maximum likelihood (ML) optimization, using a dataset of presence-absence patterns of EF-1α and EFL. One hundred optimization attempts were carried out to find the ML solution. Ancestral state probabilities were calculated using the addNode command. The reference phylogeny used for inferring patterns of gain and loss was derived from the Bayesian analysis of SSU nrDNA, *rbc*L and *atp*B, and was varied as follows. First, the majority rule consensus tree provided by MrBayes was used. Second, a rate-smoothed version of this consensus tree was used to have branch lengths roughly proportional to evolutionary time. Third, topological uncertainty was introduced in the analysis by repeating analyses on the entire post-burnin set of MCMC trees after they had been rate-smoothed. For the analysis on MCMC trees, ancestral state probabilities were calculated with the addMRCA instead of the addNode command. Rate estimates and ancestral state probabilities were averaged across the MCMC trees. We opted not to use BayesTraits' Bayesian inference because we found its output to be strongly influenced by prior settings.

In addition to these analyses, several specific hypotheses about ancestral genome content (EFL, EF-1α or both) were compared using ML optimization on the rate-smoothed reference tree. Constraints on ancestral genome content were placed on 5 ancestral nodes with the fossil command in BayesTraits, resulting in 3^5 ^= 243 hypotheses for which the log-likelihoods could be compared. Only hypotheses within two log-likelihood units from the ML solution were retained for interpretation. This set of hypotheses can be seen as a confidence set because two log-likelihood units is considered a significance threshold for such analyses [52].

The BayesTraits output was mapped onto the trees with TreeGradients v1.02 [53]. This program plots ancestral state probabilities on a phylogenetic tree as colors along a color gradient.

## Authors' contributions

EC, ODC, HV and KS designed the study. EC carried out lab work. EC and FL maintained algal cultures and performed sequence alignment. EC and HV analyzed data and drafted the manuscript. FWZ provided *atp*B sequences. All authors revised and approved the final manuscript.

## Supplementary Material

Additional file 1 **Figure S1.** Complete 72-taxon reference tree of SSU rDNA, *rbc*L and *atp*B. Click here for file

Additional file 2 **Figure S2.** Complete phylogeny inferred from amino acid sequences of EFL and related subfamilies of the GTPase translation factor superfamily. Click here for file

Additional file 3 **Figure S3.** Hypotheses about ancestral presence-absence patterns of elongation factor genes. Click here for file

Additional file 4 **Figure S4.** Hypotheses about ancestral presence-absence patterns of elongation factor genes, assuming that EF-1α is not acquired by lateral gene transfer. Click here for file

Additional file 5 **Table S1.** Algal strain information. Click here for file

Additional file 6 **Table S2.** Genbank accession numbers for nucleotide sequences of *atp*B, *rbc*L, SSU rDNA, EF-1α and EFL, newly generated sequences are in boldface. Click here for file

Additional file 7 **Alignments.** Nexus files of EF-1α, EFL, Gblocks stripped EFL and SSU-*rbc*L-*atp*B alignments. Click here for file

Additional file 8 **Additional information.** Additional information on model selection procedure, and SSU rDNA alignment and partitioning strategy. Click here for file
