# Supplementary material for: Gain and loss of elongation factor genes in green algae
Source: BMC Evol Biol. 2009 Feb 12;9:39. doi: 10.1186/1471-2148-9-39 (PMC2652445; doi:10.1186/1471-2148-9-39)
Supplement: Additional file 1 — Figure S1. Complete 72-taxon reference tree of SSU rDNA, rbcL and atpB. [file 1471-2148-9-39-S1.pdf]

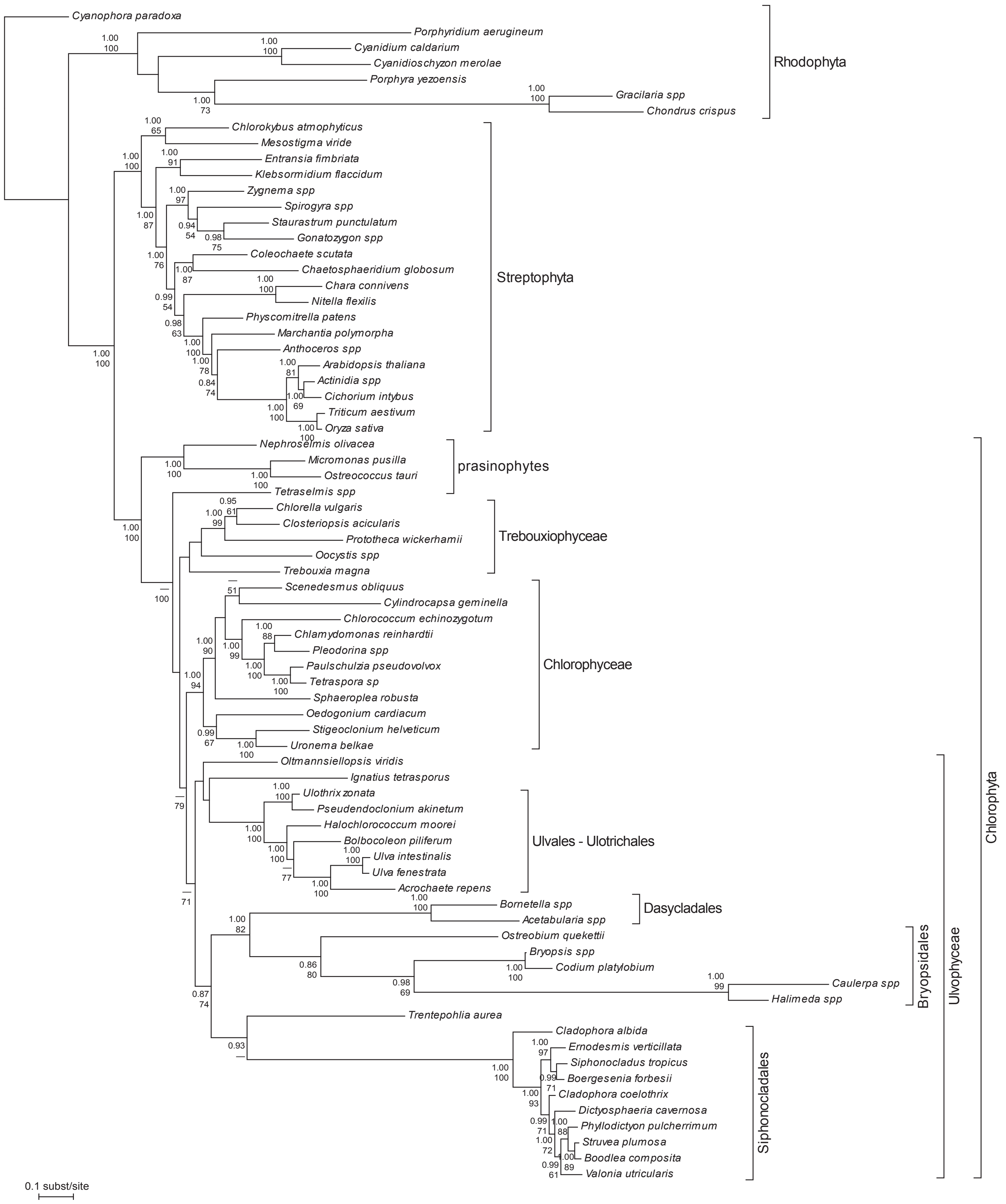

Figure S1. Reference phylogeny of the green plant lineage obtained by Bayesian inference of nuclear SSU rDNA and the plastid genes rbcL and atpB. Numbers at nodes indicate posterior probabilities (top) and ML bootstrap values (bottom); values below respectively 0.9 and 50 are not shown.
