# Supplementary material for: Gain and loss of elongation factor genes in green algae
Source: BMC Evol Biol. 2009 Feb 12;9:39. doi: 10.1186/1471-2148-9-39 (PMC2652445; doi:10.1186/1471-2148-9-39)
Supplement: Additional file 2 — Figure S2. Complete phylogeny inferred from amino acid sequences of EFL and related subfamilies of the GTPase translation factor superfamily. [file 1471-2148-9-39-S2.pdf]

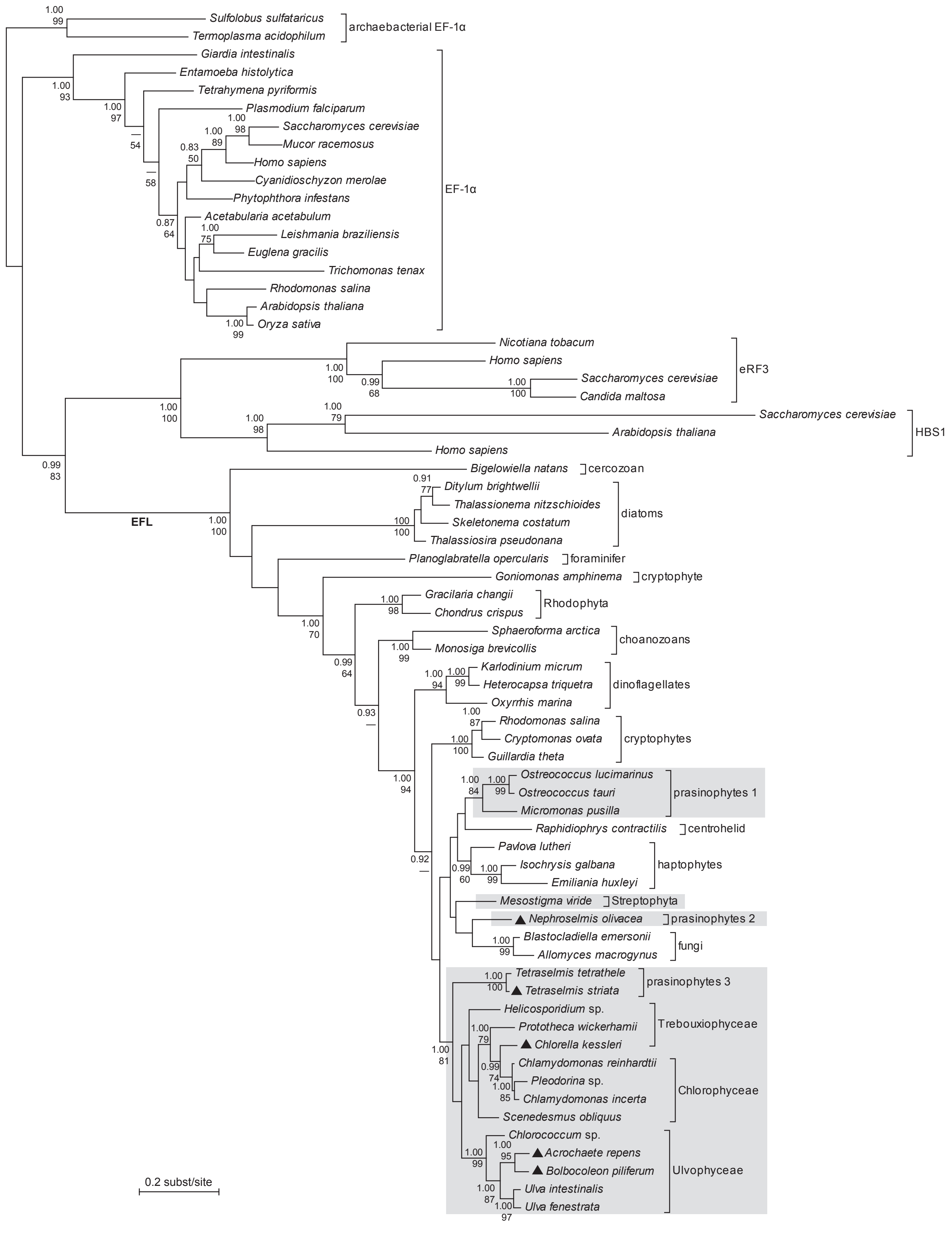

Figure S2. Phylogeny inferred from amino-acid sequences of EFL and related subfamilies of the GTPase translation factor superfamily with Bayesian techniques. Sequences belonging to the green plant lineage are in gray boxes. The green plant EFL sequences seem to form separate lineages. Numbers at nodes indicate posterior probabilities (top) and ML bootstrap values (bottom); values below respectively 0.9 and 50 are not shown.
