# Supplementary material for: Gain and loss of elongation factor genes in green algae
Source: BMC Evol Biol. 2009 Feb 12;9:39. doi: 10.1186/1471-2148-9-39 (PMC2652445; doi:10.1186/1471-2148-9-39)
Supplement: Additional file 4 — Figure S4. Hypotheses about ancestral presence-absence patterns of elongation factor genes, assuming that EF-1α is not acquired by lateral gene transfer. [file 1471-2148-9-39-S4.pdf]

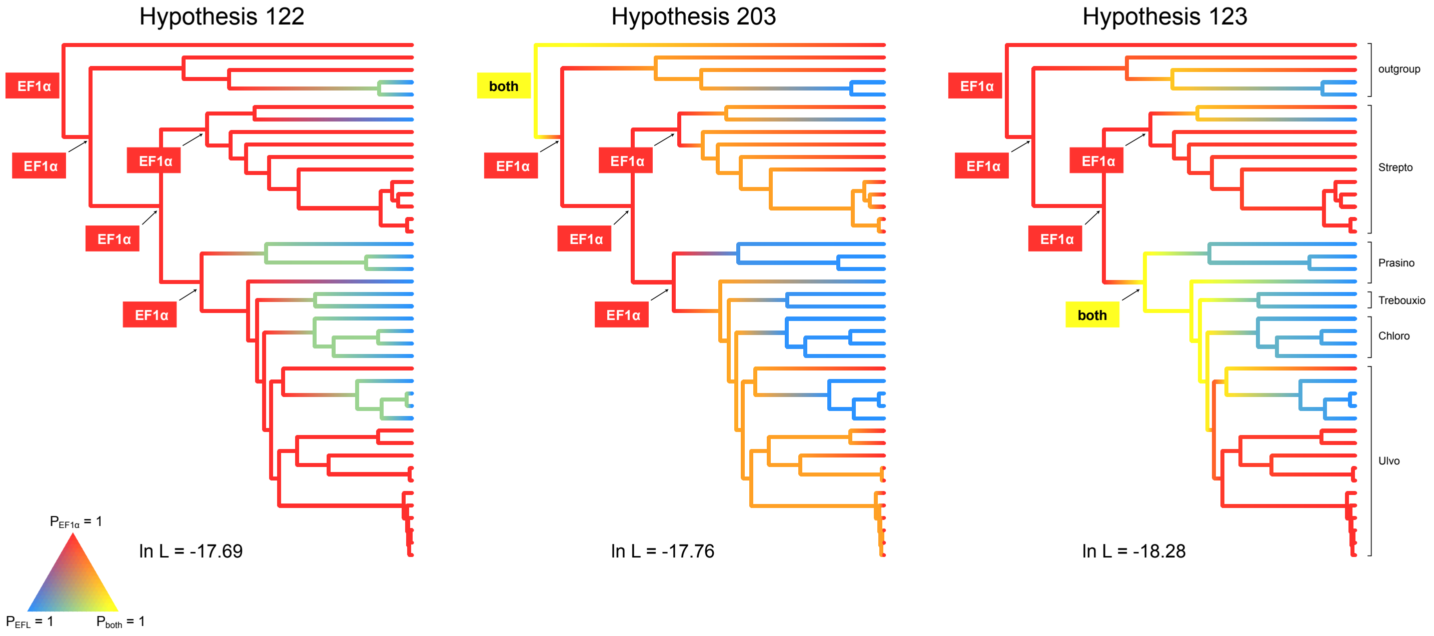

Figure S4. Hypotheses about ancestral presence-absence patterns of elongation factor genes, assuming that EF-1α is not acquired by lateral gene transfer. Likelihood scores and patterns of character evolution are visualized for variously constrained analyses. Out of the 243 tested hypotheses, only those within 2 ln L units of the ML solution are shown. The hypotheses are explained by colored boxes that indicate which nodes were constrained to have which ancestral condition. Colors were used to visualize estimated ancestral state probabilities. Red indicates a high probability for EF-1α, blue marks a high probability of EFL and yellow stands for a high probability of the presence of both genes. Intermediate colors indicate uncertainty.
