# Supplementary material for: Gain and loss of elongation factor genes in green algae
Source: BMC Evol Biol. 2009 Feb 12;9:39. doi: 10.1186/1471-2148-9-39 (PMC2652445; doi:10.1186/1471-2148-9-39)
Supplement: Additional file 5 — Table S1. Algal strain information. [file 1471-2148-9-39-S5.pdf]

	number	Culture collection
**Chlorophyta**		
**Ulvophyceae**		
**Ulvales**		
Acrochaete repens	E093db	Barbara Rinkel (Natural History Museum, London)
Bolbocoleon piliferum	E344pc	Barbara Rinkel (Natural History Museum, London)
Ulva fenestrata	EE2 and EE6	Field collection at Goese Sas (Netherlands)
Ulva intestinalis	EE3	Field collection at Goese Sas (Netherlands)
**Ignatius-clade**		
Ignatius tetrasporus	B 2012	UTEX culture collection of algae (University of Texas at Austin, USA)
**Trentepohliales**		
Trentepohlia aurea	483-1	Sammlung von Algenkulturen (University of Göttingen, Germany)
**Bryopsidales**		
Blastophysa rhizopus	LB 1029	UTEX culture collection of algae (University of Texas at Austin, USA)
Bryopsis sp.	EE4	Field collection at Goese Sas (Netherlands)
Codium sp	HEC 15711	Field collection in Madeira
Derbesia sp.	2773-1	Tatiana Klotchkova (Kongju National University, Korea)
Ostreobium quekettii	6.99	Sammlung von Algenkulturen (University of Göttingen, Germany)
**Dasycladales**		
Acetabularia acetabulum	LB 2694	UTEX culture collection of algae (University of Texas at Austin, USA)
Bornetella sphaerica	LB 2690	UTEX culture collection of algae (University of Texas at Austin, USA)
**Siphonocladales**		
Boodlea composita	Bcomp4, BoTTd75	Jeanine Olsen and Wytze Stam (University of Groningen, Netherlands)*
Cladophora coelothrix	Ccoel2, C83.14	Jeanine Olsen and Wytze Stam (University of Groningen, Netherlands)*
Dictyosphaeria cavernosa	Dcav3, D.cavSJ25b	Jeanine Olsen and Wytze Stam (University of Groningen, Netherlands)*
Ernodesmis verticillata	Erno4, EvVGa88	Jeanine Olsen and Wytze Stam (University of Groningen, Netherlands)*
Phyllodictyon orientale	Struv1, West 1631	John West (University of Melbourne, Australia)
Valonia utricularis	Vutric2, VU1546	Jeanine Olsen and Wytze Stam (University of Groningen, Netherlands)*
**Chlorophyceae**		
Chlamydomonas reinhardtii	CC1690	Chlamydomonas Center (Duke University, USA)
Scenedesmus obliquus	1450	UTEX culture collection of algae (University of Texas at Austin, USA)
**Trebouxiophyceae**		
Chlorella kessleri	211-11g	Sammlung von Algenkulturen (University of Göttingen, Germany)
**Prasinophyceae**		
Tetraselmis striata	41.85	Sammlung von Algenkulturen (University of Göttingen, Germany)
Ostreococcus tauri	OTH95	Hervé Moreau (Observatoire Océanologique de Banyuls)
Nephroselmis olivacea	40.89	Sammlung von Algenkulturen (University of Göttingen, Germany)
**Streptophyta**		
Chlorokybus atmophyticus	48.80	Sammlung von Algenkulturen (University of Göttingen, Germany)
Entransia fimbriata	LB 2793	UTEX culture collection of algae (University of Texas at Austin, USA)
Spirogyra sp.	169.80	Sammlung von Algenkulturen (University of Göttingen, Germany)

Table S1. Algal strain information.

*now maintained in the Phycology Research Group, Ghent University
