# Supplementary material for: Gain and loss of elongation factor genes in green algae
Source: BMC Evol Biol. 2009 Feb 12;9:39. doi: 10.1186/1471-2148-9-39 (PMC2652445; doi:10.1186/1471-2148-9-39)
Supplement: Additional file 6 — Table S2. Genbank accession numbers for nucleotide sequences of atpB, rbcL, SSU rDNA, EF-1α and EFL, newly generated sequences are in boldface. [file 1471-2148-9-39-S6.pdf]

	*atp*B	*rbc*L	SSU rDNA	EF-1α	EFL
**Chlorophyta**					
**Ulvophyceae**					
**Ulotrichales**					
Halochlorococcum moorei			AY198122		
Ulothrix zonata			Z47999		
**Ulvales**					
Acrochaete repens		**FJ715715**	**FJ715684**		**FJ715704**
Bolbocoleon piliferum		**FJ715716**	AY205330		**FJ715705**
Pseudendoclonium akinetum	NC 008114	NC 008114	DQ011230		
Ulva fenestrata		AF499668			EF551331/ **FJ715706**
Ulva intestinalis		AY422552	AJ000040		EF551324/ **FJ715707**
**Ignatius-clade**					
Ignatius tetrasporus			AB110439	**FJ715687**	
**Oltmannsiellopsidales**					
Oltmanssiellopsis viridis	NC 008099	NC 008099	D86495		
**Bryopsidales**					
Blastophysa rhizopus				**FJ715688**	
Bryopsis spp	FJ480417 (*B. plumosa*)	**FJ715718**	**FJ715685** (*B. plumosa*)	**FJ715689**	
Caulerpa spp	FJ480415 (*C. prolifera*)		AF479703 (*C. sertularioides*)		
Codium spp			**FJ715686** (*C. platylobium*)	**FJ715690**	
Derbesia sp.				**FJ715691**	
Halimeda spp	FJ480416 (*H. discoidea*)	**FJ715719** (*H. incrassate*)	AY786526 (*H. gracilis*)		
Ostreobium quekettii		**FJ715720**		**FJ715692**	
**Dasycladales**					
Acetabularia spp	FJ480413 (*A. dentata*)	**FJ715714** (*A. acetabulum*)	Z33461 (*A. acetabulum*)	EF551321/ **FJ715693** (*A. acetabulum*)	
Bornetella spp	FJ480414 (*B. nitida*)	**FJ715717** (*B. sphearica*)	Z33464 (*B. nitida*)	**FJ715694** (*B. sphearica*)	
**Siphonocladales**					
Boergesenia forbesii			AM498746		
Boodlea composita			AF510157	**FJ715695**	
Cladophora albida			Z35317		
Cladophora coelothrix			Z35315	**FJ715696**	
Dictyosphaeria cavernosa			AM498756	**FJ715697**	
Ernodesmis verticillata			AM498757	**FJ715698**	
Phyllodictyon spp			AF510163 (*P. pulcherrimum*)	**FJ715699**	
Siphonocladus tropicus			AM498761		
Struvea plumosa			AF510161		
Valonia utricularis			Z35323	**FJ715700**	
**Trentepohliales**					
Trentepohlia aurea		**FJ715722**	AB110783		
**Chlorophyceae**					
Chlamydomonas incerta					DQ122889
Chlamydomonas reinhardtii	NC 005353	NC 005353	M32703		DS496185/ **FJ715708**

	*atp*B	*rbc*L	SSU rDNA	EF-1α	EFL
Chlorococcum echinozygotum	EF113500	EF113430	U57698		EF551323
Cylindrocapsa geminella	EF119849	EF113434	AF387159		
Oedogonium cardiacum	EF113523	EF113458	U83133		
Paulschulzia pseudovolvox	AB014040	D86837	U83120		
Pleodorina spp	AB214424 (*P. starrii*)	D86834(*P.indica*)			AB095178 (*P.* sp.)
Scenedesmus obliquus	NC008101	NC008101	X56103		SOL00000077/ **FJ715709**
Sphaeroplea robusta	EF113536	EF113472	U73472		
Stigeoclonium helveticum	NC 008372	NC 008372	U83131		
Tetraspora sp.	EF113540	EF113477	U83121		
Uronema belkae	EF113544	EF113481	AF182821		
**Trebouxiophyceae**					
Chlorella spp	NC 001865 (*C. vulgaris*)	NC 001865 (*C. vulgaris*)	X13688 (*C. vulgaris*)		**FJ715710** (*C. kessleri*)
Closteriopsis acicularis	EF113502	EF113433	Y17470		
Helicosporidium sp.					AY729488
Oocystis spp	EF113524 (*O. apiculata*)	EF113549 (*O. apiculata*)	AF228686 (*O. solitaria*)		
Prototheca wickerhamii	AJ245645				PWL00000297
Trebouxia magna	EF113541	AJ969630	Z21552		
**Prasinophyceae**					
**Chlorodendrales**					
Tetraselmis spp	DQ173248 (*T. suecica*)	DQ173247 (*T. suecica*)	X70802 (*T. striata*)		EF551330 (*T. tetrathele*)<br>**FJ715711** (*T. striata*)
**Mamiellales**					
Micromonas pusilla		AY955031	AJ010408		EF551325
Ostreococcus lucimarinus					XM 001420948
Ostreococcus tauri	NC 008289	NC 008289	AY329635		CR954213/ **FJ715713**
**Pseudoscourfieldiales**					
Nephroselmis olivacea	NC 00927	NC 00927	X74754		**FJ715712**
**Streptophyta**					
**Mesostigmatales**					
Mesostigma viride	NC 002186	NC 002186	AJ250109		DQ394295
**Chlorokybales**					
Chlorokybus atmophyticus	DQ422812	DQ422812	M95612	**FJ715701**	
**Klebsormidiales**					
Entransia fimbriata	AY823688	E88 slordig	LB2793	**FJ715702**	
Klebsormidium flaccidum	AF408801	E87 rbcL2	X75520		
**Zygnematales**					
Gonatozygon spp	AF408796 (*G. monotaenium*)	U71438 (*G. monotaenium*)	X91346 (*G. aculeatum*)		
Spirogyra spp	AF408797 *S. maxima*	**FJ715721** (*S.* sp.)		EF551328/ **FJ715703**	
Staurastrum punctulatum	NC 008116	NC 008116	AF115442		
Zygnema spp	NC 008117 (*Z. circumcarin*)	NC 008117 (*Z. circumcarin*)	AJ853450 (*Z. pseudogedeanum*)		
**Coleochaetales**					
Chaetosphaeridium globosum	NC 004115	NC 004115	AF113506		

	*atp*B	*rbc*L	SSU rDNA	EF-1α	EFL
Coleochaete scutata	AY082303	AY082313	X68825		
**Charales**					
Chara spp	AF408782 (*C. connivens*)	L13476 (*C. connivens*)	U18493 (*C. connivens*)	EF551322 (*C. australis*)	
Nitella flexilis	AB110837	AB076056	U05261		
**Embryophyta (land plants)**					
**Anthoceratophyta (Hornworts)**					
Anthoceros spp	NC 004543 (*A. formosa*)	NC 004543 (*A. formosa*)	X80984 (*A. agrestis*)		
**Marchantiophyta (Liverworts)**					
Marchantia polymorpha	NC 001319	NC 001319	AB021684		
**Bryophyta (Mosses)**					
Physcomitrella patens	AP005672	AB066207		XM 001753007	
**Spermatophyta (Seed plants)**					
Actinidia spp	AJ235382 (*A. chinensis*)	L01882 (*A. chinensis*)	U42495 (*A.* sp.)	AY946009 (*A. deliciosa*)	
Arabidopsis thaliana	NC 000932	NC 000932	AC006837	AK230352	
Cichorium intybus		L13652		AY378166	
Oryza sativa	AC092750	AJ746297	X00755	AF030517	
Triticum aestivum	NC 002762	NC 002762	AY049040	M90077	
Vicia faba				AJ222579	
**other eukaryotes**					
**Archaeplastida**					
**Glaucocystophyta**					
Cyanophora paradoxa	NC 001675	NC 001675	AY823716	AF092951	
**Rhodophyta**					
**Bangiophyceae**					
Porphyra yezoensis	AP006715		D79976	U08844	
Porphyridium aerugineum			AJ421145		
**Cyanidiophyceae**					
Cyanidioschyzon merolae	AB002583		AB158485	AB095182	
Cyanidium caldarium	X66698		AB091232		
**Florideophyceae**					
Chondrus crispus		CCU02984	Z14140		CO652990 - CO652099- CO652788
Gracilaria spp	AY673996 (*G. tenuistipitata*)	AY673996 (*G. tenuistipitata*)	L26210 (*G. verrucosa*)		DV963090 - DV963156 - DV964877 (*G. changii*)
**Chromalveolates**					
**Apicomplexans**					
Plasmodium falciparum				X60488	
**Ciliates**					
Tetrahymena pyriformis				D11083	
Tetrahymena thermophila				XM 001032213	
**Cryptophytes**					
Goniomonas amphinema					AB332031
Guillardia theta					AM183813

	*atp*B	*rbc*L	SSU rDNA	EF-1α	EFL
Rhodomonas salina					DQ659244
**diatoms**					
Ditylum brightwellii					AB368772
Phaeodactylum tricornutum				18475 (JGI, v2.0)	
Skeletonema costatum					AB368773
Thalassionema nitzschioides					AB368774
Thalassiosira pseudonana				3858 (JGI, v3.0)	41829 (JGI, v3.0)
**Dinoflagellates**					
Heterocapsa triquetra					AY729485
Karlodinium micrum					EF134135
Oxyrrhis marina					DQ659243
**Haptophytes**					
Emiliania huxleyi					CV068986
Isochrysis galbana					AY729486
Pavlova lutheri					AY729487
**Heterokonts**					
**Oomycetes**					
Phytophthora infestans				AJ249839	
**Opisthokonts**					
**Fungi**					
Allomyces macrogynus					EC637105
Blastocladiella emersonii					EF064246
Mucor racemosus				MRATEF1A	
Saccharomyces martiniae				AF402021	
**Metazoa (Animals)**					
Homo sapiens				NM 001958	
**choanoflagellates**					
Monosiga brevicollis					XM_001745603
Sphaeroforma arctica					DQ403164
**Excavates**					
**Diplomonads**					
Giardia intestinalis				D14342	
Giardia lamblia				XM 76292	
**Euglenids**					
Euglena gracilis				X16890	
**Parabasalids**					
Trichomonas tenax				D78479	
Trichomonas vaginalis				XM 001325448	
**Kinoplastids**					
Leishmania braziliensis				XM 001563727	
**Amoebozoa**					
Entamoeba histolytica				ENHEF1ALPH	

	*atp*B	*rbc*L	SSU rDNA	EF-1α	EFL
**Rhizaria**					
**Cercozoa**					
Bigelowiella natans					AY729489
**Foraminifera**					
Planoglabratrella opecularis					AB334123
**Centrohelids**					
Raphidiophrys contractilis					AB332032

Table S2. GenBank accession numbers or DOE Joint Genome Institute (JGI) transcript identifiers for nucleotide sequences of *atp*B, *rbc*L, SSU rDNA, EF-1α and EFL. Newly generated sequences are in boldface. GenBank accession numbers for protein sequences of eRF3 (*Candida maltosa* BAB12681, *Homo sapiens* NP_002085, *Nicotiana tabacum* AAA79032, *Saccharomyces cerevisiae* EDN60510), HBS1 (*Arabidopsis thaliana* NP_196625, *Homo sapiens* NP_006611, *Saccharomyces cerevisiae* NP_01301) and archaebacterial EF-1α (*Sulfolobus sulfataricus* CAA50033, *Termoplasma acidophilum* P19486) are given here between brackets.
