# Supplementary material for: Gain and loss of elongation factor genes in green algae
Source: BMC Evol Biol. 2009 Feb 12;9:39. doi: 10.1186/1471-2148-9-39 (PMC2652445; doi:10.1186/1471-2148-9-39)
Supplement: Additional file 8 — Additional information. Additional information on model selection procedure, and SSU rDNA alignment and partitioning strategy. [file 1471-2148-9-39-S8.pdf]

SSU rDNA alignment and partitioning

The SSU rDNA sequences were aligned on the basis of their rRNA secondary structure information using the following procedure. The SSU rDNA sequences of several green plant representatives incorporated in the European Ribosomal RNA Database (Wuyts et al. 2004, http://www.psb.ugent.be/rRNA/) were used as an initial model for building the alignment. The alignment editor DCSE v2.6 (De Rijk and De Wachter, 1993) was used to annotate and check the secondary structure, and manually align the sequences. For the newly generated sequences, the alignment of the highly variable helices 43 and 49 [see De Rijk et al. (1999) for secondary structure nomenclature of the SSU gene] was refined and aided by folding the RNA sequences using mfold v.3.2 with default temperature and RNA parameters (Zuker 2003, http://mfold.bioinfo.rpi.edu/). Finally, we used the Xstem software (Telford et al., 2005) to extract the RNA secondary structure information from DCSE to a nexus format with stem/loop partitions.

Model selection procedure

Selection of a suitable partitioning strategy and suitable models for the partitions followed a three-step procedure and uses the Akaike Information Criterion (AIC) as a selection criterion. The guide tree used during the entire procedure was obtained by MP analysis of the concatenated data using PAUP* 4.0b10 (Swofford 2003). All subsequent likelihood optimizations and AIC calculations were carried out with Treefinder (Jobb et al. 2004). The first step consisted of optimizing the likelihood for nine potential partitioning strategies, assuming a HKY+G8 model for each partition. The three partitioning strategies with the lowest AIC scores (i.e., those providing the best fit to the data) were retained for further evaluation. The second step involved model selection for individual partitions. The likelihood of each partition present in the three retained partitioning strategies was optimized for three variants of the general time reversible model (F81, HKY and GTR), with and without inclusion of a discrete gamma distribution (eight categories) to model among-site rate heterogeneity. Because not all genes were sampled for all taxa, the guide tree was pruned to the taxa present in the partition in question before each optimization. The partition-specific models obtaining the lowest AIC score were passed on to the third step, which consisted of re-evaluation of the three partitioning strategies retained from the first step using the models selected for these partitions in the second step. The partitioning strategy + model combination that received the lowest AIC score in the third step was used in the phylogenetic analyses documented in the main text.

References

De Rijk P, De Wachter R. 1993. DCSE, an interactive tool for sequence alignment and secondary structure research. Computer Applications in the Biosciences 9:735-740.

De Rijk P, Robbrecht E, de Hoog S, Caers A, Van de Peer Y, De Wachter R. 1999. Database on the structure of large subunit ribosomal RNA. Nucleic Acids Research 27:174-178.

Jobb G, von Haeseler A, Strimmer K. 2004. TREEFINDER: a powerful graphical analysis environment for molecular phylogenetics. Bmc Evolutionary Biology 4.

Telford MJ, Wise MJ, Gowri-Shankar V. 2005. Consideration of RNA secondary structure significantly improves likelihood-based estimates of phylogeny: Examples from the Bilateria. Molecular Biology and Evolution 22:1129-1136.

Swofford DL. 2003. PAUP*. Phylogenetic Analysis Using Parsimony (*and Other Methods). Version 4. Sinauer Associates, Sunderland, Massachusetts.

Wuyts J, Perriere G, Van de Peer Y. 2004. The European ribosomal RNA database. Nucleic Acids Research 32:D101-D103.

Zuker M. 2003. Mfold web server for nucleic acid folding and hybridization prediction. Nucleic Acids Research 31:3406-3415.
